# Modulation of Endocannabinoid-Binding Receptors in Human Neuroblastoma Cells by Tunicamycin

**DOI:** 10.3390/molecules24071432

**Published:** 2019-04-11

**Authors:** Cinzia Rapino, Annalisa Castellucci, Anna Rita Lizzi, Annalaura Sabatucci, Clotilde B. Angelucci, Daniel Tortolani, Gianna Rossi, Gabriele D’Andrea, Mauro Maccarrone

**Affiliations:** 1 Faculty of Veterinary Medicine, Agriculture and Environment, University of Teramo, 64100 Teramo, Italy; crapino@unite.it (C.R.); bcangelucci@unite.it (C.B.A.); dtortolani@unite.it (D.T.); 2 Department of Biotechnology and Applied Clinical Sciences, University of L’Aquila, 67100 L’Aquila, Italy; annacastel@libero.it (A.C.); annarita.lizzi@cc.univaq.it (A.R.L.); gabriele.dandrea@cc.univaq.it (G.D.); 3 Faculty of Biosciences and Technology for Food, Agriculture and Environment, University of Teramo, 64100 Teramo, Italy; alsabatucci@unite.it; 4 Department of Life, Health and Environmental Sciences, University of L’Aquila, 67100 L’Aquila, Italy; gianna.rossi@cc.univaq.it; 5 Department of Medicine, Campus Bio-Medico University of Rome, 00128 Rome, Italy; 6 European Center for Brain Research, IRCCS Santa Lucia Foundation, 00164 Rome, Italy

**Keywords:** SH-SY5Y cells, endocannabinoid-binding receptors, tunicamycin, *N*-acetylglucosamine

## Abstract

Endocannabinoid (eCB)-binding receptors can be modulated by several ligands and membrane environment, yet the effect of glycosylation remains to be assessed. In this study, we used human neuroblastoma SH-SY5Y cells to interrogate whether expression, cellular localization, and activity of eCB-binding receptors may depend on *N*-linked glycosylation. Following treatment with tunicamycin (a specific inhibitor of *N*-linked glycosylation) at the non-cytotoxic dose of 1 µg/mL, mRNA, protein levels and localization of eCB-binding receptors, as well as *N*-acetylglucosamine (GlcNAc) residues, were evaluated in SH-SY5Y cells by means of quantitative real-time reverse transcriptase-polymerase chain reaction (qRT-PCR), fluorescence-activated cell sorting (FACS), and confocal microscopy, respectively. In addition, the activity of type-1 and type-2 cannabinoid receptors (CB_1_ and CB_2_) was assessed by means of rapid binding assays. Significant changes in gene and protein expression were found upon tunicamycin treatment for CB_1_ and CB_2_, as well as for GPR55 receptors, but not for transient receptor potential vanilloid 1 (TRPV1). Deglycosylation experiments with *N*-glycosidase-F and immunoblot of cell membranes derived from SH-SY5Y cells confirmed the presence of one glycosylated form in CB_1_ (70 kDa), that was reduced by tunicamycin. Morphological studies demonstrated the co-localization of CB_1_ with GlcNAc residues, and showed that tunicamycin reduced CB_1_ membrane expression with a marked nuclear localization, as confirmed by immunoblotting. Cleavage of the carbohydrate side chain did not modify CB receptor binding affinity. Overall, these results support *N*-linked glycosylation as an unprecedented post-translational modification that may modulate eCB-binding receptors’ expression and localization, in particular for CB_1_.

## 1. Introduction

Type-1 (CB1) and type-2 (CB2) cannabinoid receptors belong to the seven-transmembrane G protein-coupled receptors (GPCRs) family [1]. They are pivotal components of the endocannabinoid system [2], through which endocannabinoids (eCBs) exert many of their effects both centrally [3] and peripherally [4]. Accumulated evidence suggests the presence of additional receptor targets for eCBs on the cell surface, such as the GPR55 receptor [5], and the transient receptor potential vanilloid 1 (TRPV1) ion channel [6]. The activation of these receptors by eCBs triggers several pathways that control distinct physiologic processes [3,4], as well as a wide range of neurodegenerative and neuroinflammatory disorders such as Alzheimer’s disease, Parkinson’s disease, amyotrophic lateral sclerosis, and multiple sclerosis [7,8].

Most, but not all, GPCRs are known to be *N*-linked glycoproteins with heterogeneous oligosaccharides, e.g., D2, D3 dopamine receptors [9], β1-adrenergic receptors [10], nicotinic acetylcholine receptors [11], and A2 adenosine receptors [12]. Of note, available data on GPCRs have shown that glycosylation is a post-translational modification influencing receptor expression and processing, ligand binding, and/or coupling to second messengers [9,13].

In the case of tumor cells, glycosylation is dramatically altered during disease progression due to changes in the expression levels or activity of glycosyltransferases and glycosidases [14,15,16]. Several reports have shown a crucial role for *N*-linked carbohydrates also in cell-cycle progression and cell viability [17]. These modifications have been associated with enhanced malignancy, and thus they could profoundly impact the modulation of tumor growth [18]. For instance, neuroblastoma, which accounts for 10% of childhood cancers, exhibits aberrant cell-surface glycosylation patterns [19]. Specific glycosylation inhibitors are widely used to interrogate the role of glycosylation in various biological processes, including protein folding and conformation, oligomerization, sorting, cell-cell interactions, and targeting of proteins to sub- or extra-cellular locations [17]. In this context, tunicamycin acts as a specific inhibitor of *N*-linked glycosylation, and blocks the first step of glycoprotein synthesis, i.e., the UDP-*N*-acetylglucosamine-dolichol phosphate *N*-acetylglucosamine-1-phosphate transferase (GPT); thus, it arrests the synthesis of all *N*-linked glycoproteins [20]. Consequently, there will be an accumulation of misfolded or unfolded glycoproteins in the endoplasmic reticulum (ER), leading to ER stress. Indeed, it has been shown that in many cell types, ER stress can be induced by treating cells with low concentrations of tunicamycin [21]. Instead, at higher concentrations, tunicamycin promotes prostate cancer cell death by activating the mammalian target of rapamycin complex-1 (mTORC1)-dependent pathway [22]. On the other hand, the process of glycosylation is extremely sensitive and can be inhibited by small amounts of tunicamycin, as reported in mouse podocytes and human embryonic kidney (HEK-293) cells [23]. Recently, tunicamycin has been shown to impair also phosphorylation of Anaplastic Lymphoma Kinase (ALK), thus disrupting pro-survival signaling in neuroblastoma cells [24]. In this investigation, the effect of tunicamycin on the major eCB-binding receptors (namely, CB_1_, CB_2_, GPR55, and TRPV1) was interrogated in human neuroblastoma SH-SY5Y cells, which indeed express all of them [25]. The aim was to ascertain whether glycosylation can regulate their expression and subcellular distribution, and therefore signal transduction thereof.

## 2. Results

### 2.1. Effects of Tunicamycin on mRNA and Protein Expression of eCB-Binding Receptors

**Figure 1:**
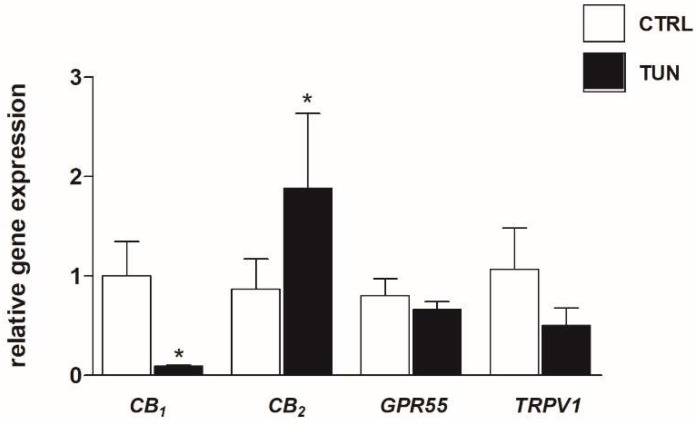
Effect of tunicamycin on mRNA expression of endocannabinoid (eCB)-binding receptors. SH-SY5Y cells were treated for 24 h with 1 µg/mL tunicamycin (TUN). Data are presented as means ± SEM (n = 3). [* *p* < 0.05 vs. control cells (CTRL)].

The mRNA expression of CB_1_, CB_2_, *GPR55*, and *TRPV1* was evaluated in SH-SY5Y cells upon treatment for 24 h with tunicamycin at the non-cytotoxic dose of 1 µg/mL, by means of qRT-PCR (Figure 1). Tunicamycin concentration was chosen after investigating, by Trypan blue exclusion test, the effects of various doses on cell viability (data not shown). As shown in Figure 1, CB_1_ mRNA expression decreased significantly (*p* < 0.05 vs. control) following tunicamycin treatment. In contrast, CB_2_ mRNA levels were significantly increased (*p* < 0.05 vs. control) after exposure to tunicamycin (Figure 1), whereas *GPR55* expression was unaffected and *TRPV1* expression showed a trend towards decrease, though not statistically significant (Figure 1).

Then, the effect of tunicamycin on eCB-binding receptor protein expression was assessed in SH-SY5Y cells under the same experimental conditions through FACS analysis. The intracellular quantitation of eCB-binding receptors, calculated as mean fluorescence intensity values (MFI), revealed a significant decrease of CB_1_ and GPR55 in cells exposed to 1 µg/mL tunicamycin (*p* < 0.001 for CB_1_; *p* < 0.05 for GPR55) (Table 1). Instead, CB_2_ and TRPV1 expression slightly increased after tunicamycin treatment (Table 1). In addition, to further corroborate the efficacy of tunicamycin, SH-SY5Y cells were challenged with an anti-biotin-WGA antibody that specifically binds to GlcNAc. As expected, tunicamycin-treated samples showed a significant decrease in WGA expression (*p* < 0.001 vs. control) (Table 1).

### 2.2. Assessment of N-linked Glycosylation after Tunicamycin Treatment

All eCB-binding receptors under investigation (CB_1_, CB_2_, GPR55 and TRPV1) showed potential *N*-glycosylation sites, as documented by their sequence analysis through the UNIPROT database (Table 2). Incidentally, such a post-translational modification (PTM) is characterized by a glycosidic bond between GlcNac and a nitrogen atom (usually N4) of an Asn residue in a consensus sequence Asn-X-Ser/Thr (where X is any aminoacid except Pro)—more rarely Asn-X-Cys. In particular, CB_1_ presents three putative glycosylated Asn residues, all located in the N-terminal region, whereas CB_2_ and TRPV1 receptors have only one putative glycosylation site, and GPR55 has two (Table 2).

**Figure 2:**
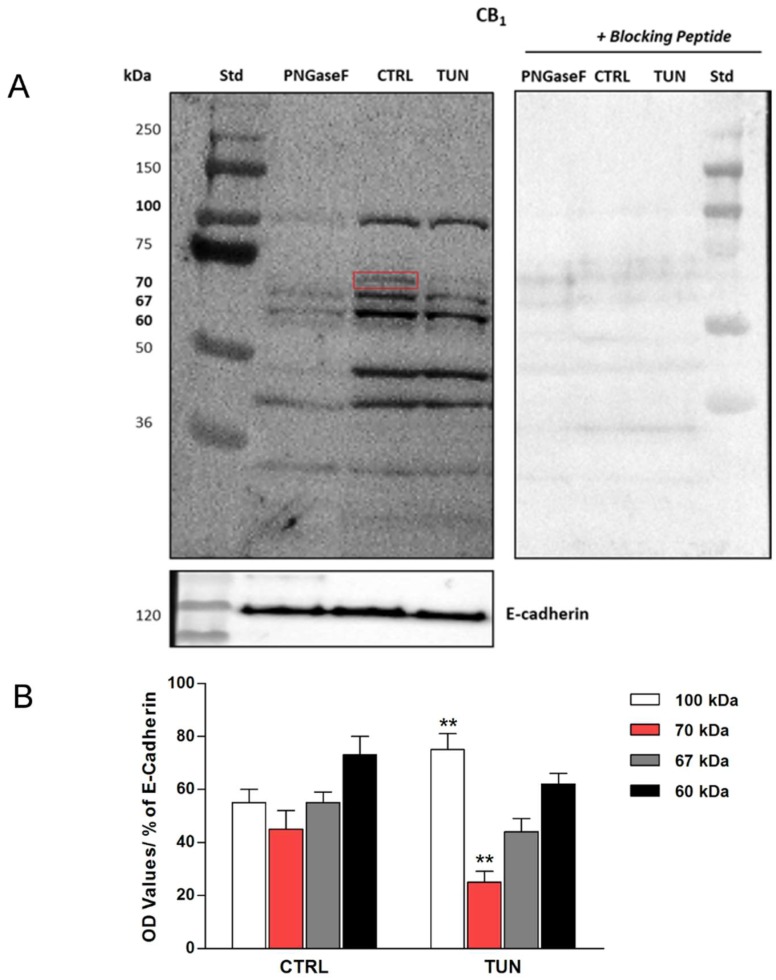
Representative Western blot of tunicamycin effect on cannabinoid receptor 1 (CB_1_) protein expression. SH-SY5Y cells were treated for 24 h with tunicamycin (TUN 1 µg/mL) and with PNGase F (20 µL of 500 U/mL solution), as indicated. Controls with specific blocking peptides are also shown. The red square indicates the immunoreactive band at 70 kDa, erased by tunicamycin and PNGase F treatments (**A**). Densitometric analysis of CB_1_ immunoreactive bands (100 kDa, 70 kDa, 67 kDa and 60 kDa) normalized to E-cadherin content. Data are presented as means ± SEM (n = 3) [** *p* < 0.01 vs. CTRL] (**B**).

CB_1_ was the most affected eCB-binding receptor by tunicamycin treatment, both at mRNA and protein levels, and had a greater extent of glycosylation compared to other receptors. Thus, its electrophoretic mobility in plasma membranes isolated from SH-SY5Y cells, after treatment with tunicamycin and PNGaseF (which removes all N-linked sugars) was evaluated by means of Western blot analysis. First, to ascertain the specificity of anti-CB_1_ antibody, antigen preabsorption experiments were carried out with the corresponding blocking peptide. In particular, the latter erased different immunoreactive bands (at 60 kDa, 67 kDa, 70 kDa, and 100 kDa) which could correspond to CB_1_ forms with different glycosylation motifs, and to a dimeric form of the receptor (Figure 2A). Upon treatment of the cells with tunicamycin, the immunoreactive band at 70 kDa almost disappeared, suggesting receptor deglycosylation (Figure 2A). In particular, densitometric analysis of immunoreactive bands revealed a significant ~55% reduction of the 70 kDa band in tunicamycin-treated samples compared to the control samples (*p* < 0.01 vs. control) (Figure 2B). This finding was confirmed by PNGase F treatment, which also erased the 70 kDa band (Figure 2A). Moderate reductions (i.e., ~20% and ~15%) of the intensity of both 67 kDa, and 60 kDa bands were shown in tunicamycin-treated samples compared to controls (Figure 2B), whereas the band at 100 kDa appeared to be more significantly expressed (~40%) in the same samples than in controls (*p* < 0.01 vs. control) (Figure 2B). Incidentally, no changes were detected by Western blot for CB_2_, GPR55, and TRPV1 (data not shown).

### 2.3. Localization of eCB-Binding Receptors in SH-SY5Y Cells upon Tunicamycin Treatment

**Figure 3:**
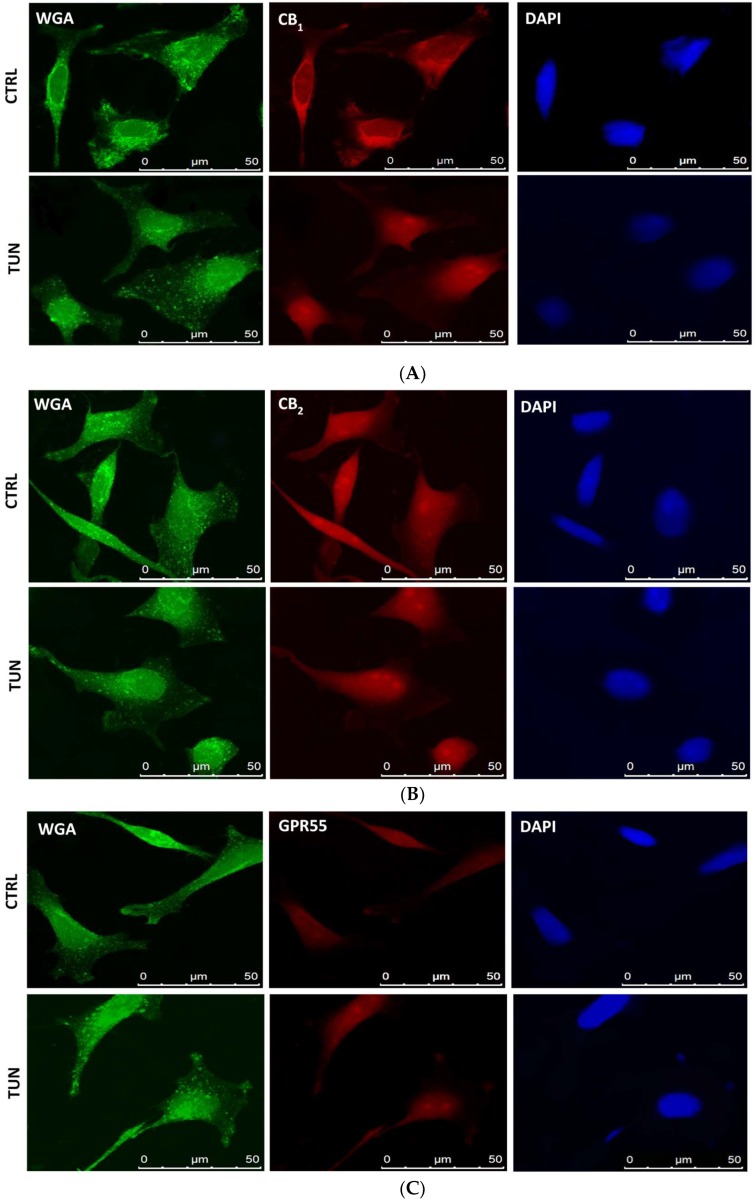

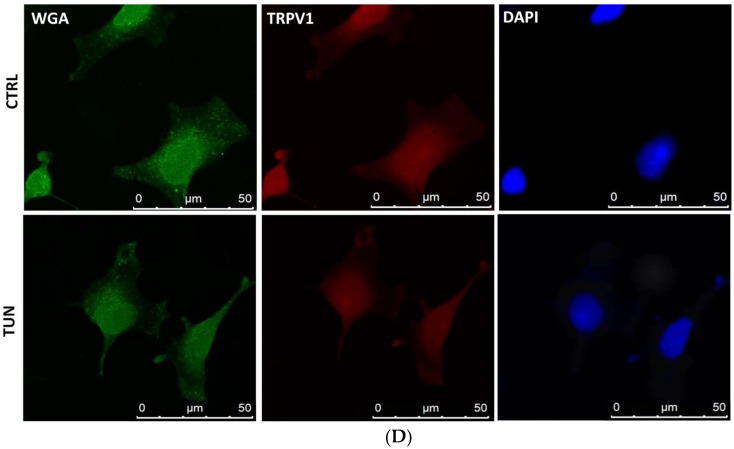
Co-localization of eCB-binding receptors with *N*-acetylglucosamine (GlcNAc). Representative triple immunofluorescence staining of GlcNAc (green), eCB-binding receptors (red), and DAPI (blue) in SH-SY5Y cells. SH-SY5Y cells were incubated with a biotinylated lectin (WGA) to visualize total GlcNAc, and with specific antibodies [anti-CB_1_ (**A**), anti-CB_2_ (**B**), anti-GPR55 (**C**), and anti-TRPV1 (**D**)] for eCB-binding receptors; then with Alexa Fluor 488-labelled streptavidine for GlcNAc residues, with anti-rabbit IgG labeled with Alexa Fluor 595 for eCB-binding receptors and with DAPI for DNA. Scale bars = 50 μm.

**Figure 4:**
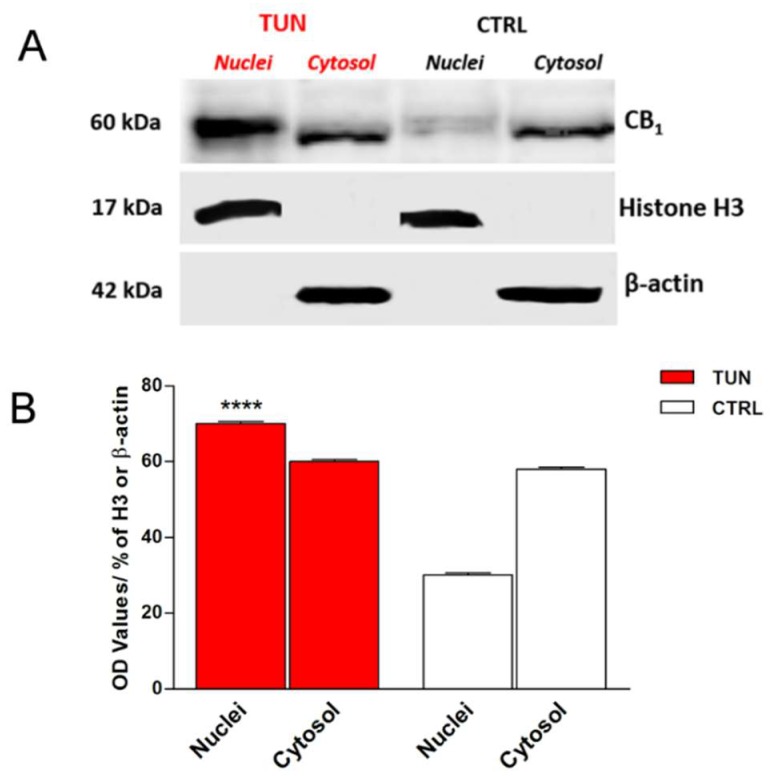
CB_1_ immunostaining in the nuclear and cytoplasmatic fractions of SH-SY5Y cells, treated or not for 24 h with tunicamycin (TUN 1 µg/mL). Histone H3 and β-actin were used as loading controls for nuclear and cytoplasmatic fractions (**A**). Densitometric analysis of CB_1_ immunoreactive band normalized to histone H3 and β-actin. Data are presented as means ± SEM (n = 3) [**** *p* < 0.0001 vs. nuclei CTRL] (**B**).

In order to demonstrate that some of eCB-binding receptors were indeed glycosylated, their co-localization with GlcNAc residues was assayed in the SH-SY5Y cells by means of confocal analysis. Subcellular localization of CB_1_ revealed a diffuse fluorescence throughout the cytoplasm and membranes, and overlapped with biotin-WGA staining (Figure 3A). After tunicamycin treatment, CB_1_ immunostaining was predominant in the nucleus with sparse co-localization with WGA (Figure 3A). To confirm these data, cell fractionation followed by immunoblotting experiments was performed in SH-SY5Y cells upon tunicamycin treatment. CB_1_ was found to be more abundant in the nucleus than in the cytoplasm of treated cells (Figure 4A), and consistently densitometric analysis showed a significant 40% higher CB_1_ immunoreactive band in the nuclei of treated cells than in control cells (*p* < 0.0001 vs. control) (Figure 4B). Instead, CB_2_ staining was almost completely cytosolic, with scarce or absent association to the plasma membrane (Figure 3B). No co-localization with WGA was observed for CB_2_ and GPR55, both under control conditions and upon tunicamycin treatment (Figure 3B,C). Finally, a faint overlap between TRPV1 and WGA staining was detected on cell membranes of controls and tunicamycin-treated cells (Figure 3D).

### 2.4. Effects of Tunicamycin on CB ligand Binding

In order to determine whether CB receptors were functionally affected by tunicamycin, a pan-CB ligand binding assay was performed in SH-SY5Y cells treated for 24 h with 1 µg/mL tunicamycin. Binding of the synthetic cannabinoid [^3^H]CP55940, a CB_1_ and CB_2_ agonist [26] was substantially unchanged in tunicamycin-treated cells with respect to controls (Table 3), suggesting that tunicamycin treatment did not affect CB receptor function in SH-SY5Y cells.

## 3. Discussion

Glycosylation is well-known to regulate surface expression of GPCRs, like β_1_ adrenergic receptor [10] and D_2_ receptor [9]. Based on in silico data, also eCBs receptors have putative *N*-glycosylation sites [27,28], yet at present, it remains unclear whether the latter sites are indeed glycosylated in real life. Human CB_1_ and CB_2_ receptors appear to differ in the number and distribution of their potential *N*-glycosylation sites. In the *N*-terminal region, CB_2_ has only one potential *N*-glycosylation site, whereas CB_1_ has three of them. Two potentially glycosylated Asn residues are conserved in rat and mouse species (Asn 77 and Asn 83) [28]. Moreover, the human CB_1_ sequence has two splice variants (hCB_1_a and hCB_1_b) that differ at their *N*-terminus. In particular, hCB_1_b shows a deletion of 33 amino acids that includes Asn 77 and Asn 83, and remarkably this variant has been shown to play a role in metabolic regulation [29]. Recently, two splice variants resembling those of the human receptor were discovered also in the mouse CB_1_-encoding gene *CNR1* [30]. Here, the lack of *N*-glycosylation sites was found to strongly reduce glycosylation level and mitogen-activated protein kinase (MAPK) activity upon CB_1_ agonist-induced stimulation [30]. In this context, it should be recalled that preliminary data on tunicamycin treatment of CB_1_ failed to show any efficacy on the inhibition of downstream cyclic AMP accumulation in cultured mouse neuroblastoma N18TG2 cells, suggesting that glycosylation was not engaged in this CB_1_-dependent signaling pathway [31]. Yet, in the same investigation, the authors cautioned that no agonist binding data were obtained to correlate receptor activation with signal transduction thereof [31]. On the other hand, tunicamycin has already been shown to modify CB_2_ protein expression profiles in methylotrophic yeast *Pichia pastoriis,* where a glycosylation site at the *N*-terminus of the receptor was demonstrated, and the carbohydrate portion accounted for ~3 kDa [32]. As for TRPV1, the presence of *N*-glycosylation in rats was first shown in 2001 [33], and shortly after, its functional role was reported [34]. Indeed, it was found that *N*-glycosylation may affect basic functional characteristics of TRPV1, representing a major determinant of capsaicin-evoked desensitization and ionic permeability [34,35]. Conversely, little information (if any) is available on the presence of *N*-glycosylation sites in GPR55, though sequence analysis via the UNIPROT database strongly supports it at the *N*-terminus as a PTM of the expressed protein. Up to date the question of whether *N*-glycosylation sites are essential for eCB-binding receptors function remains largely unanswered. At any rate, our results seem to suggest that glycosylation exerts distinct effects on different eCB-binding receptors, extending previous studies on other GPCRs [10,36,37]. Interestingly, removal of the sugar moiety led to a decreased expression of CB_1_, resembling previous data on rat EP3 prostaglandin receptor, human AT1 angiotensin-II receptor, human 5-HT5A serotonin receptor, human B2 bradykinin receptor, human TXA2 thromboxane receptor, and human D5 dopamine receptor [38,39,40,41,42]. In the case of rat EP3β-subtype PGE_2_ receptor, glycosylation appeared to be essential also for an efficient translocation to the plasma membrane [43]. It seems noteworthy that tunicamycin is a commonly used ER stressor that induces the unfolded protein response (UPR) by activating specific ER protein signaling, which in turn leads to inflammatory processes [44]. In this context, CB_2_ is known to have protective actions in different chronic inflammatory diseases [45]. Therefore, here it might be speculated that the increased gene CB_2_ expression induced by tunicamicyn may be a compensatory response that cooperates with UPR in re-establishing cellular homeostasis. However, this merely speculative hypothesis remains to be ascertained in independent studies. Notably, tunicamycin has increased expression of GPR55 receptor, whereas no effect was evident on TRPV1, as demonstrated by flow cytometry and quantified as mean fluorescence intensity values (MFI). 

Remarkably, treatment with tunicamycin has revealed the presence of at least one glycosylated form in CB_1_ but not in the other eCB-binding receptors under investigation (data not shown), as demonstrated by the shift of the immunoreactive band at 70 kDa. This is supported by the fact that the extracellular amino-terminal part of CB_1_ contains three consensus sequences (Asn77, Asn83, and Asn112) that suitable for *N*-glycosylation. On the other hand, PNGase F treatment seems to confirm the presence of glycosylated forms in CB_1_, because the molecular weight of this receptor decreased when deglycosylated. In addition, WGA is a useful tool for detecting glyconjugates on cell membrane [46]. Co-localization of this molecule with CB_1_ confirmed the presence of sugar residues on CB_1_. Interestingly, tunicamycin seemed to affect also the cellular distribution of CB_1_, but not of the other eCB-binding receptors analyzed. Indeed, CB_1_ was found to be more localized to the nucleus in tunicamycin-treated cells than in controls. This finding is in line with a recent study, showing that glycosylation is important for cytoplasmic retention of estrogen receptor GPR30 [47]. 

On a final note, here we demonstrated that tunicamycin has no effect on CB_1_/CB_2_ binding activity, although it can be anticipated that independent site-directed mutational studies are deemed necessary to further our understanding of the functional significance of *N*-glycosylated residues in these two cannabinoid receptors. 

In conclusion, this study supports the concept that, although all major eCB-binding receptors could be potentially glycosylated in human neuroblastoma cells, the role of such a post-transcriptional modification (PTM) differs from receptor to receptor. In the case of CB_1_, glycosylation appears necessary for normal receptor expression and localization. Therefore, it should be added to other PTMs recently reported to regulate CB_1_, such as palmitoylation of its cysteine 415 [48], and interaction with membrane cholesterol [49]. Incidentally, so far it has been shown that only *N*-glycosylated isoforms of Neurotensin receptor-1 (NTSR-1), a GPCR that has been identified as a mediator of cancer progression, are able to localize with membrane structured microdomains by palmitoylation for efficient mitogenic signaling [50]. It will be interesting to further investigate whether in CB_1_ there is a similar interdependence between palmitoylation and glycosylation, also in the light of the development of novel therapeutic strategies to combat CB_1_-dependent diseases in humans [51,52]. 

## 4. Materials and Methods 

### 4.1. Materials

Dulbecco’s modified Eagle’s medium, fetal calf serum were from Gibco (Life Technologies, Grand Island, NY, USA). Biotinylated labeled wheat germ agglutinin (WGA), bisbenzimide Hoechst 33,258 (H33258), tunicamycin, protease inhibitor, peptide *N*-glycosidase (PNGase F) from Elizabethkingia miricola and all the other reagents were from Sigma-Aldrich Co (St. Louis, MO, USA). Streptavidine conjugate labeled with Alexa Fluor 488 was from Molecular Probes (Eugene, Oregon, USA). Antibody antirabbit IgG conjugate labeled with Alexa Fluor 595 was purchased from Life Technologies (Life Technologies, Grand Island, NY, USA).

### 4.2. Cell Culture

Human neuroblastoma SH-SY5Y cells were grown in Dulbecco’s modified Eagle’s medium, supplemented with 15% inactivated fetal bovine serum, 2 mM l-glutamine, 100 units/mL penicillin/streptomycin, 1 mM sodium pyruvate, 1 mM Hepes, and 1 mM nonessential amino acids [25]. Cells were maintained at 37 °C in a humidified 5% CO_2_ atmosphere at a density of 2 × 10^5^ cells/mL and were treated with different amounts (1, 2 or 3 µg/mL) of tunicamycin, or with vehicle (DMSO, CTRL), for 24 h. Cells were counted and viability was determined by Trypan blue exclusion assay [25].

### 4.3. Quantitative Real Time-Reverse Transcriptase-Polymerase Chain Reaction (qRT-PCR) Analysis

Total mRNA was extracted from SH-SY5Y cells by using TRIzol (Life technologies, Grand Island, NY, USA) according to the manufacturer instructions. Quantification of total mRNA samples was assessed by using Thermo Scientific NanoDrop 2000c UV-Vis spectrophotometer at 260 nm (Waltham, MA, USA). cDNA was synthetized from 1 µg of total RNA of each sample by using the RevertAid H Minus First Strand cDNA Synthesis Kit (Thermo Scientific, Waltham, MA, USA). The relative abundance was assessed by RT quantitative PCR (RT-qPCR) using SensiFAST_TM_ SYBR Lo-ROX kit (Bioline, London, UK) to by adjusting the manufacturer instruction final volume of 15 µL on a 7500 Fast Real-time PCR System (Life Technologies, Grand Island, NY, USA). To provide precise quantification of the initial target in each PCR reaction, the amplification plot was examined, as well as the point of early log phase of product accumulation defined by assigning a fluorescence threshold above background, defined as the threshold cycle number or Ct. The relative expression of different amplicons was calculated by the delta–delta Ct (DDCt) method and converted to relative expression ratio (2^-DDCt^) for statistical analysis [53]. All data were normalized to the endogenous reference genes β-actin, GAPDH, and 18S rRNA combined. The primers used for PCR amplification are reported in Table 4.

### 4.4. Fluorescence-Activated Cell Sorting (FACS) Analysis

Control and treated SH-SY5Y cells (1 × 10^6^/mL) were collected, washed twice with ice-cold PBS, fixed in 4% paraformaldehyde in Phosphate-buffered saline (PBS), and permeabilized with a blocking solution solution (80 mM PIPES, 0.5% BSA, 5 mM EGTA, 1 mM MgCl_2_, 50 mM NH_4_Cl, 0.05% saponin, 0.02% (*w*/*v*) and NaN_3_, pH 6.8). *N*-acetylglucosamine (GlcNAc) and eCB-binding receptors were visualized by incubating cells with biotinylated lectin (WGA 20 µg/mL, 1 h), and with anti-CB_1_ (1:200), anti-CB_2_ (1:200), anti-GPR55 (1:200) and anti-TRPV1 (1:100) specific antibodies. After three washes with PBS, biotinylated lectin was localized with streptavidine conjugate labeled with Alexa Fluor 488, and eCB-binding receptors were identified using rabbit IgG conjugate labeled with Alexa Fluor 595. Cell fluorescence distribution was analyzed by flow cytometry (FACScan, Becton Dickinson, Mountain View, CA, USA), equipped with a CELLQuest Software Program. One thousand cells from each sample were computed, and mean fluorescence intensity (MFI) was calculated.

### 4.5. Cellular Fractionation

Subcellular fractionation was performed as reported [54]. Briefly, cell lysates were obtained by homogenization of the samples (control and treated SH-SY5Y cells) in ice-cold buffer (50 mM Tri/HCl pH 7.4) with the addition of 5 mM MgCl_2_, 250 mM sucrose and protease inhibitor cocktail and were homogenized at 10.000 rpm with RW 16 Basic homogeniser with a Teflon pestle (IKA-Werke GmbH & Co. KG, Staufen im Breisgau, Germany). Then, they were centrifuged at 800× *g* for 20 min at 4 °C. The pellet was used for the isolation of the nuclei, while the supernatant was used to isolate the cytosolic fraction. The pellet was washed with the same buffer and then centrifuged at 500× *g* for another 20 min. Cytosolic fraction was extracted by centrifugation of the supernatant at 14,000× *g* for 30 min at 4 °C. Nuclei were suspended in ice-cold buffer (20 mM Hepes pH 7.9 with the addition of 1.5 mM MgCl_2_, 0.5 M NaCl, 0.2 mM EDTA, 20% glycerol, and 1% Triton-X-100 and protease inhibitor cocktail) and sonicated twice (amplitude 40, cycle 1) in dry ice. The number of proteins was determined by the Bio-Rad Protein assay (Bio-Rad Laboratories, Hemel Hempstead, UK).

### 4.6. Protein Deglycosylation Assay and Immunoblotting 

Cell lysates were obtained by homogenization of the samples (control and treated SH-SY5Y cells) in a PBS ice-cold buffer with the addition of 1 mM MgCl_2_, 1 mM CaCl_2_, and 1 mM DTT and were sonicated twice (amplitude 40, cycle 1) in dry ice. Next, they were centrifuged at 1000× *g* for 10 min at 4 °C, and the supernatant was collected and centrifuged at 20,000× *g* for 30 min at 4 °C. The pellet (cell membranes) was recovered and resuspended in 50 mM Tris/HCl buffer pH7.5. The amount of proteins was determined by the Bio-Rad Protein assay (Bio-Rad Laboratories, Hemel Hempstead, UK). Control Samples for deglycosylation studies were treated with 20 μL PNGase F (500 U/mL) of *N*-glycosidase F in 60 mM sodium phosphate pH 7.5, 0.35% SDS, 70 mM DTT and 1% Triton x-100. The reaction mixture was incubated for 18 h at 37 °C and was stopped by cooling at 4 °C before the addition of the sample buffer for immunoblotting analysis. Equal amounts of total extracts (30 μg of protein) were electrophoresed on 10% or 12% acrylamide gels, then gels were electroblotted overnight onto 0.45 µm nitrocellulose membranes (Biorad Laboratories, CA, USA). Membranes, nuclei, and cytoplasmatic fractions were saturated with a solution of 3% BSA, then were incubated with anti-CB_1_ (1:200) (Cayman Chemicals, Ann Arbor, MI, USA, item n. 101500) and with polyclonal antibodies for Histone H3 (PA5-16183, ThermoFisher Scientific, Rockford, USA, 1:20,000), cadherin 1 (ABIN1440031, Antibodies-online GmbH, Germany, 1:500), and monoclonal antibody for β-actin (8457, Cell Signaling Technology, Leiden, The Netherlands, 1:1000) for nuclei, membrane, and cytosolic fractions respectively. To block the formation of the antibody/protein complex, the blocking peptides for CB_1_ (Cayman Chemicals, Ann Arbor, MI, USA, item n. 301500) was preincubated in a 1:1 ratio (*v*/*v*) with the corresponding antibody for 1 h at room temperature, then it was diluted to the final working antibody concentration. Goat anti-rabbit-HRP (1:10,000, Thermo Fisher Scientific, MA, USA) was used as secondary antibody. Detection was performed by using the West Dura Chemiluminescence System (Pierce, Rockford, IL, USA). Blots were developed using the LiteAblot Plus Enhanced Chemiluminescent substrate (Euroclone S.p.A, Milano, Italy). The intensity of the immunoreactive bands was quantified by densitometric analysis through the ImageJ software (NIH, Bethesda, MD, USA).

### 4.7. Confocal Microscopy

Control and treated SH-SY5Y cells (2 × 10^5^/mL) were cultured for 24 h and then fixed in 4% paraformaldehyde. Immunofluorescence staining was performed using a biotinylated lectin (WGA) 20 µg/mL diluted in blocking solution for 1 h to visualize total *N*-acetylglucosamine and with specific antibodies for eCB-binding receptors [1:200 for CB_1_, CB_2_ and GPR55 (Cayman Chemicals, Ann Arbor, MI, USA, item n. 101500, n. 101550, n. 10224), 1:100 for TRPV1 (Santa Cruz Biotechnology Inc., Santa Cruz, CA, sc-12498)] in blocking solution for 1 h. Cells were washed with PBS and incubated with streptavidine labeled with Alexa Fluor 488 for *N*-acetylglucosamine residues and with anti-rabbit IgG labelled with Alexa Fluor 595 in order to identify all eCB receptors. Cells were washed again with PBS and counterstained with H33258 (0.5 µg/mL) for 5 min at RT. After three more washes, samples were resuspended in a MOWIOL solution, placed on a slide, and examined at a Leica TCS SP5 II DMI6000 confocal microscope (Leica Microsystems, Mannheim, Germany) equipped with HCX plan apo 63¥ (numerical aperture 1.4) oil immersion objective. Fluorescent images were derived from the maximum projection of optical serial sections (step size 0.29 μM) using the LAS AF software (2.6.0.7266, Leica Microsystems). For presentation purposes, LAS AF pictures were exported in TIFF format and processed with Adobe Photoshop CS5 (Mountain View, CA, USA) for adjustments of brightness and contrast. 

### 4.8. Receptor Binding Assay on Adherent Living Cells

Control and treated SH-SY5Y cells (1.5 × 10^5^/well) were cultured in a 12-well cell culture plate. After tunicamycin treatment for 24 h, each well was washed twice with 1 mL of PBS and treated with 500 μL of incubation buffer (50 mM Tris–HCl, 5 mM MgCl_2_, 1 mM CaCl_2_, 0.2% BSA, pH 7.4), preheated to 37 °C, in the presence of 1 μM “cold” CP55.940 and incubate for 15 min at 37 °C. Then, 2.5 nM [3H]CP55.940 was added and incubated for 1 h in an incubator set at 37 °C. After incubation, the buffer was carefully removed and cells were washed again with ice-cold washing buffer (50 mM Tris–HCl, 500 mM NaCl, 0.1% BSA, pH 7.4). Then, 500 μL of 0.5 M NaOH was added to each well, and cells were pipetted up and down several times to lyse them. The resuspension was then transferred to a 10 mL scintillation vial with liquid scintillation cocktail, and immediately read radioactivity in a scintillation β-counter (Tri-Carb 2810 TR, Perkin Elmer, Waltham, MA, USA) [55]. 

### 4.9. Statistical Analysis

Data are reported as means ± S.E.M or S.D. of at least three independent experiments, each performed in duplicate. Data were analyzed by the Prism 5 program (GraphPad Software, La Jolla, CA, USA), using unpaired *t*-test and one-way or two-way analysis of variance (ANOVA) followed by Tukey test or Bonferroni post hoc analysis, as appropriate. A level of *p* < 0.05 was considered statistically significant.

## Figures and Tables

**Table 1:** Mean fluorescent intensity (MFI) values of SH-SY5Y cells expressing eCB-binding receptors, and GlcNAc residues. * Denotes *p* < 0.001; ** *p* < 0.05.

Experimental Group	CB_1_	CB_2_	GPR55	TRPV1	GlcNAc
CTRL	70.60 ± 2.12	16.70 ± 0.50	30.00 ± 0.90	19.30 ± 0.60	146.00 ± 4.38
TUN	55.80 ± 1.67 *	20.40 ± 0.61	22.60 ± 0.68 **	20.40 ± 0.61	131.40 ± 3.94 *

**Table 2:** Potential *N*-glycosylation sites detected by sequence analysis of human eCB-binding receptors in the Uniprot database (www.uniprot.org).

Receptor	UNIPROT Entry	Putative Glycosylated Asn Residues	Asn Position
CB_1_	P21554	3	77, 83, 112
CB_2_	P34972	1	11
GPR55	Q9Y2T6	2	5, 171
TRPV1	Q8NER1	1	604

**Table 3:** CB_1_ and CB_2_ binding activity of control SH-SY5Y cells, and of cells treated with tunicamycin (TUN 1 µg/mL) for 24 h.

Experimental Group	CB Binding Activity (pmol/mg of Protein)
CTRL	17.40 ± 1.79
TUN	19.87 ± 0.36

Data are presented as means ± SEM (n = 3).

**Table 4:** List of primer sequences used for qRT-PCR analysis.

Human Gene	Forward (5′→3′)	Reverse (3′→5′)
CB_1_	CCTTTTGCTGCCTAAATCCAC	CCACTGCTCAAACATCTGAC
CB_2_	TCAACCCTGTCATCTATGCTC	AGTCAGTCCCAACACTCATC
*GPR55*	ATCTACATGATCAACCTGGC	ATGAAGCAGATGGTGAAGACGC
*TRPV1*	TCACCTACATCCTCCTGCTC	AAGTTCTTCCAGTGTCTGCC
*β-Actin*	TTCTACAATGAGCTGCGTG	AGAGGCGTACAGGGATAGCA
*GAPDH*	GATTCCACCCATGGCAAATTC	TGGGATTTCCATTGATGACAAG
*18S rRNA*	CGCCGCTAGAGGTGAAATTCT	CGAACCTCCGACTTTCGTTCT
